# Homeostatic mechanisms in dopamine synthesis and release: a mathematical model

**DOI:** 10.1186/1742-4682-6-21

**Published:** 2009-09-10

**Authors:** Janet A Best, H Frederik Nijhout, Michael C Reed

**Affiliations:** 1 Department of Mathematics, The Ohio State University, Columbus, OH 43210, USA; 2 Department of Biology, Duke University, Durham, NC 27708, USA; 3 Department of Mathematics, Duke University, Durham, NC 27708, USA

## Abstract

**Background:**

Dopamine is a catecholamine that is used as a neurotransmitter both in the periphery and in the central nervous system. Dysfunction in various dopaminergic systems is known to be associated with various disorders, including schizophrenia, Parkinson's disease, and Tourette's syndrome. Furthermore, microdialysis studies have shown that addictive drugs increase extracellular dopamine and brain imaging has shown a correlation between euphoria and psycho-stimulant-induced increases in extracellular dopamine [1]. These consequences of dopamine dysfunction indicate the importance of maintaining dopamine functionality through homeostatic mechanisms that have been attributed to the delicate balance between synthesis, storage, release, metabolism, and reuptake.

**Methods:**

We construct a mathematical model of dopamine synthesis, release, and reuptake and use it to study homeostasis in single dopaminergic neuron terminals. We investigate the substrate inhibition of tyrosine hydroxylase by tyrosine, the consequences of the rapid uptake of extracellular dopamine by the dopamine transporters, and the effects of the autoreceoptors on dopaminergic function. The main focus is to understand the regulation and control of synthesis and release and to explicate and interpret experimental findings.

**Results:**

We show that the substrate inhibition of tyrosine hydroxylase by tyrosine stabilizes cytosolic and vesicular dopamine against changes in tyrosine availability due to meals. We find that the autoreceptors dampen the fluctuations in extracellular dopamine caused by changes in tyrosine hydroxylase expression and changes in the rate of firing. We show that short bursts of action potentials create significant dopamine signals against the background of tonic firing. We explain the observed time courses of extracellular dopamine responses to stimulation in wild type mice and mice that have genetically altered dopamine transporter densities and the observed half-lives of extracellular dopamine under various treatment protocols.

**Conclusion:**

Dopaminergic systems must respond robustly to important biological signals such as bursts, while at the same time maintaining homeostasis in the face of normal biological fluctuations in inputs, expression levels, and firing rates. This is accomplished through the cooperative effect of many different homeostatic mechanisms including special properties of tyrosine hydroxylase, the dopamine transporters, and the dopamine autoreceptors.

## Background

Dopamine is a catecholamine that is used as a neurotransmitter both in the periphery and in the central nervous system (CNS)[2-4]. Important nuclei that contain dopaminergic neurons include the substantia nigra pars compacta and the ventral tegmental area [5]. These nuclei send projections to the neostriatum, the limbic cortex, and other limbic structures [3].

Dopamine is known to play an important role in many brain functions. Dopamine affects the sleep-wake cycle [6], it is critical for goal-directed behaviors [7] and reward-learning [8], and modulates the control of movement via the basal ganglia [9,10]. Cognitive processing, such as executive function and other pre-frontal cortex activities, are known to involve dopamine [11]. Finally, dopamine contributes to synaptic plasticity in brain regions such as the striatum and the pre-frontal cortex [12-14].

Dysfunction in various dopaminergic systems is known to be associated with various disorders. Reduced dopamine in the pre-frontal cortex and disinhibited striatal dopamine release is seen in schizophrenic patients [15]. Loss of dopamine in the striatum is a cause of the loss of motor control seen in Parkinson's patients [16]. Studies have indicated that there is abnormal regulation of dopamine release and reuptake in Tourette's syndrome [17]. Dopamine appears to be essential in mediating sexual responses [18]. Furthermore, microdialysis studies have shown that addictive drugs increase extracellular dopamine and brain imaging has shown a correlation between euphoria and psycho-stimulant-induced increases in extracellular dopamine [1]. These consequences of dopamine dysfunction indicate the importance of maintaining dopamine functionality through homeostatic mechanisms that have been attributed to the delicate balance between synthesis, storage, release, metabolism, and reuptake [19,20]. It is likely that these mechanisms exist both at the level of cell populations [21,22] and at the level of individual neurons.

In this paper we construct a mathematical model of dopamine synthesis, release, and reuptake and use it to study homeostasis in single dopaminergic neuron terminals. It is known that the enzyme tyrosine hydroxylase (TH), the rate limiting enzyme in dopamine synthesis, has the unusual property of being inhibited by its own substrate, tyrosine [23]. Cytosolic dopamine concentrations are normally quite low because most dopamine resides in vesicles from which it is released on the arrival of action potentials. After release, dopamine is rapidly taken up by dopamine transporters (DATs) on the terminal and it is thought that the DATs play an important role in extracellular dopamine homeostasis [24,25]. Autoreceptors are found on most parts of dopaminergic neurons, in particular the neuron terminal. It was first proposed in the 1970's [26,27] that the binding of dopamine to presynaptic autoreceptors affects TH and therefore the synthesis of dopamine. It is now known that increased extracellular dopamine can inhibit TH by at least 50% [28,29] and the data in [30], [31], and [32] suggest that when extracellular dopamine drops, synthesis can be increased by a factor of 4 to 5. The purpose of our modeling is to tease apart the contributions of these various mechanisms to the homeostasis of dopamine synthesis, release, and reuptake.

**Figure 1:**
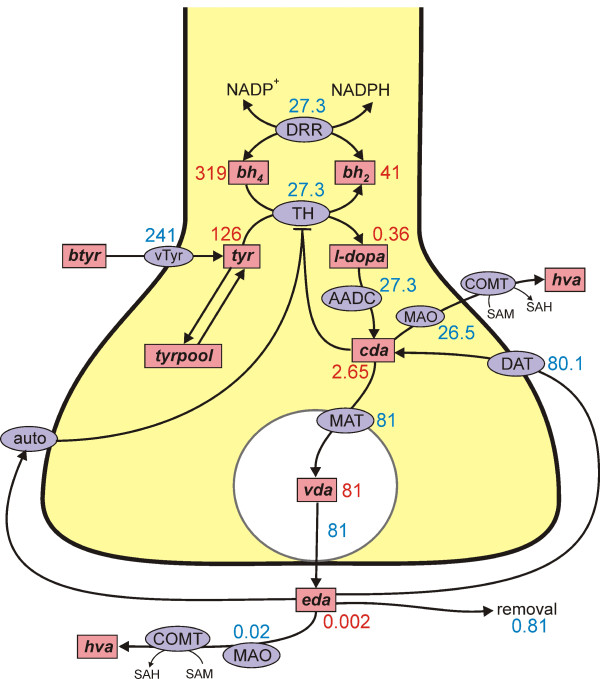
**Dopamine synthesis, release, and reuptake**. The figure shows the reactions in the model. Rectangular boxes indicate substrates and blue ellipses contain the acronyms of enzymes or transporters. The numbers indicate the steady state concentrations (*μ*M) and reaction velocities (*μ*M/hr) in the model. Full names for the substrates are in Methods. Other acronyms: vTyr, neutral amino acid transporter; DRR, dihydrobiopterin reductase; TH, tyrosine hydroxylase; AADC, aromatic amino acid decarboxylase; MAT, vesicular monoamine transporter; DAT, dopamine transporter; auto, D2 dopamine auto receptors; MAO monoamine oxidase; COMT, catecholamine O-methyl transferase.

A schematic diagram of the model is indicated in Figure 1. The pink boxes contain the acronyms of substrates and the blue ellipses the acronyms of enzymes and transporters; full names are give in the Methods. Dopamine is synthesized in the nerve terminal from tyrosine which is transported across the blood brain barrier. We include exchange between tyrosine and a tyrosine pool that represents all the other uses and sources of tyrosine in the terminal. Tyrosine is converted into L-3,4-dihydroxyphenylalanine (*l-dopa*) by tyrosine hydroxylase (TH) and *l-dopa *is converted into cytosolic dopamine (*cda*) by aromatic amino acid decarboxylase (AADC). Cytosolic dopamine is transported into the vesicular compartment by the monoamine transporter and vesicular dopamine (*vda*) is released from the vesicular compartment into the extracellular space at a rate proportional to the firing rate of the neuron. In the extracellular space, extracellular dopamine (*eda*) affects the autoreceptors, is taken up into the terminal by the DATs and is removed from the system by uptake into glial cells and the blood and diffusion out of the striatum. Dopamine is also catabolized both in the terminal and in the extracellular space.

There have been a number of other models of dopamine dynamics. Ours is closest in spirit to the quite comprehensive model by Justice [33] based on experimental work by Justice, Michael and others [34-36]. They did not consider fluctuations in blood tyrosine or intracellular tyrosine nor did they consider the effects of autoreceptors. The model by Porenta and Riederer [37] is less detailed but does include the effects of autoreceptors. Tretter and Eberie [38] have a very simple model of behavior at the synapse. Nicholson [39] studied the difficult mathematical questions involved in diffusion and reuptake of dopamine in extracellular spaces with realistic irregular geometry. Qi *et al*. [40,41] use a general modeling framework in which the rates of change of all variables are written as sums of powers of the other variables and then coefficients and exponents are determined by fitting data. Kaushik et al. [42] focus on the regulation of TH by phosphorylation, iron, and *α*-synuclein. Fuente-Fernandez et al. [43] created a probabilistic model of synthesis and release to see if stochastic variation could cause the motor fluctuations in Parkinson's disease. Wightman and co-workers use models of release into and reuptake from the extracellular space to infer properties of the DATs and to interpret their data on the time courses of extracellular dopamine [44-47]. They added diffusion in the extracellular space in [48] and used the model and their experiments to show that the concentration of DA is quite uniform in the extracellular space during tonic firing but not during burst firing.

We use the mathematical model as a platform on which to investigate the system effects of variations in quantities such as enzyme expression levels, tyrosine inputs, firing rate changes, and concentrations of dopamine transporters. We find that dopaminergic function is under tight regulatory control so that the system can respond strongly to significant biological signals such as bursts, but responds only moderately to the normal noisy fluctuations in the component parts of the system.

## Methods

The mathematical model consists of nine differential equations for the variables listed in Table 1. We denote substrates in lower case so that they are easy to distinguish from enzyme names and velocities, which are in upper case. Reaction velocities or transport velocities begin with a capital V followed by the name of the enzyme, the transporter, or the process as a subscript. For example, *V*_TH_(*tyr, bh*4, *cda, eda*) is the velocity of the tyrosine hydroxylase reaction and it depends on the concentrations of its substrates, *tyr *and *bh*4, as well as *cda *(end product inhibition), and *eda *(via the autoreceptors). Below we discuss in detail the more difficult modeling issues and reactions with non-standard kinetics. Table 2 gives the parameter choices and references for reactions that have Michaelis-Menten kinetics in any of the following standard forms:



**Table 1:** Variables

bh2	dihydrobiopterin
bh4	tetrahydrobiopterin
tyr	tyrosine
l-dopa	3,4-dihyroxyphenylalanine (L-DOPA)
cda	cytosolic dopamine
vda	vesicular dopamine
eda	extracellular dopamine
hva	homovanillic acid
tyrpool	the tyrosine pool

**Table 2:** Kinetic Parameters (*μ*M, *μ*M/hr,/hr).

**velocity**	**parameter**	**model value**	**literature value**	**references**
*V*_AADC_	aromatic amino acid decarboxylase			
	*K*_*m*_	130	130	[94]
	*V*_*max*_	10,000		*
*V*_DAT_	dopamine transporter			
	*K*_*m*_	.2	0.2-2	[75,76]
	*V*_*max*_	8000		*
*V*_DRR_	dihydropteridine reductase			
	*K*_*bh*2_	100	4-754	[95,96]
	*K*_NADPH_	75	29-770	[70-80,97-99]
		200		*
	*K*_*bh*4_	10	1.1-17	[100,98]
	*K*_NADP_	75	29-770	[70-80,97-99]
		80		*
*V*_MAT_	vesicular monoamine transporter			
	^*K*^*m*	3	.2-10	[101-103]
	^*V*^*max*	7082		*
	^*k*^*out*	40		*
*V*_TH_	tyrosine hydroxylase			
	*K*_*tyr*_	46	46	[60]
	*K*_*bh*4_	60	13,	[60]
	*V*_*max*_	125		*
	*K*_*i *_(cda)	110	110	[104]
	*K*_*i *_(substrate inhibition)	160	46	[23,60] ; 160
	*K*_*i *_(autoreceptors)			*
*V*_TYRin_	neutral amino acid transporter			
	*K*_*m*_	64	64	[51]
	*V*_*max*_	400		*
*tyr *↔ *tyrpool*				
	*k*_1_	6		*
	*k*_-1_	0.6		*
catabolism and diffusion				
		0.2		*
		10		*
		30		*
		3	3.3	[68]
		3.45	3.45	[69,70]
		0.2		*
	*k*_*rem*_	400		*

* see text

for unidirectional, one substrate, unidirectional, two substrates, and bidirectional, two substrates, two products, respectively.

Table 1 gives the abbreviations used for the variables throughout. The differential equations corresponding to the reactions diagramed in Figure 1 follow.



### Tyrosine and the tyrosine pool

A wide range of tyrosine concentrations, 39-180 *μ*M, have been measured in serum in infants and adults [49,50], with means near 100 *μ*M. In our model we take the serum concentration to be *btyr *= 97 *μ*M. In the model experiments described in Results A, this concentration varies throughout the day due to meals but averages 97 *μ*M. Tyrosine is transported from the serum across the blood-brain barrier (BBB) to the extracellular space and from there into the neuron. We simplify this two-step process into a single step from the serum into the neuron with velocity *V*_TYRin _and assume that the kinetics are those of the neutral amino acid transporter across the BBB. The *K*_*m *_of the transporter has been measured as 64 *μ*M [51] and we take *V*_*max *_= 400 *μ*M/hr, so



If *btyr *has its average value of 97 *μ*M, then *V*_TYRin _= 244 *μ*M/hr, which corresponds almost exactly to the 4 *μ*M/min reported in [51] for the import of tyrosine into the brain.

Intracellular tyrosine is used in a large number of biochemical and molecular pathways and is produced by many pathways [52]. Over 90% of the tyrosine that enters the intracellular pool of the brain is used in protein synthesis [53-55] and even in the striatum a relatively small fraction is used for dopamine synthesis [55]. To represent all of the other products and sources of tyrosine, we will use a single variable *tyrpool*, and assume that it exchanges linearly with the tyrosine pool:



We choose the rate constants *k*_1 _= 6 *μ*M/hr and *k*_-1 _= 0.6 *μ*M/hr so that *tyrpool *is approximately 10 time larger than *tyr*. As we will see below, with this choice, about 10% of the imported tyrosine goes to dopamine synthesis and the steady state tyrosine concentration is 126 *μ*M in the model, well within the normal range of 100-150 *μ*M [56]. The importance of *tyrpool *is that, without it, all imported tyrosine would have to go to dopamine in the model. Not only would that be incorrect physiologically, but dopamine synthesis would be extremely sensitive to tyrosine import, which it is not [57,58,56].

### Tyrosine hydroxylase

Tyrosine (*tyr*) and tetrahydrobiopterin (*bh*4) are converted by tyrosine hydroxylase (TH) into 3,4-dihyroxyphenylalanine (*l-dopa*) and dihyrobiopterin (*bh*2). The velocity of the reaction, *V*_*TH*_, depends on *tyr*, *bh*4, cytosolic dopamine (*cda*), and extracellular dopamine (*eda*) via the autoreceptors:



The third term (on the right side of the equation) is simply Michaelis-Menten kinetics including the inhibition of TH by *cda *which competes with *bh*4 [3,59,23]. Values for the rate constants and references are given in Table 2. The first term (on the right) is substrate inhibition of the enzyme by tyrosine itself [23]. A range of values for *K*_*i*(*tyr*)_, 37-74 *μ*M, was found in [60]. We have computed *K*_*i*(*tyr*) _= 160 *μ*M directly from the data in figure 2 of [23]. The number 0.56 in the numerator is chosen so that at steady state the overall value of this term is one. That means the the steady states with and without substrate inhibition will be the same and this will allow us to make comparisons of the the dynamic behaviors of the TH reaction in the two cases (Results A).

**Figure 2:**
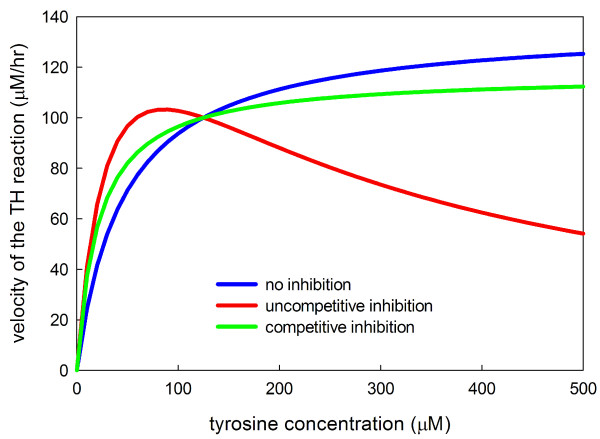
**Michaelis-Menten and substrate inhibition kinetics**. The three curves plot the velocity of the TH reaction as a function of the concentration of tyrosine for normal Michaelis-Menten kinetics, for competitive substrate inhibition, and for uncompetitive substrate inhibition. The curves have been normalized so that each has velocity 100 *μ*M/hr when the tyrosine concentration is 125 *μ*M. In each case *K*_*m *_= 46 *μ*M.

The second term (on the right) requires more discussion. It was first proposed in the 1970's [26,27] that the binding of dopamine to presynaptic autoreceptors affects TH and therefore the synthesis of dopamine. Although the details of the mechanisms are not certain, research since that time has demonstrated clearly that the autoreceptors modulate the activity of TH as well as the neuronal firing rate and the release of dopamine[29,28,61,64,31]. All three effects are consistent: higher *eda *means more stimulation of the autoreceptors and this decreases the activity of TH [29,63], lowers firing rate [61,62], and inhibits release [28,29]. The evidence in these papers suggests that dopamine agonists can inhibit TH by at least 50% [28,29]. The more difficult question is how much synthesis is increased if the normal inhibition by the autoreceptors is released? In [63] only a 40% increase was found, but the data in [30] and [31] suggest that synthesis can be increased by a factor of 4 to 5. This is consistent with the original data in [27], Table 1. The third factor in the formula for *V*_TH_(*tyr, bh*4*, cda, eda*) has the following properties: at the normal steady state it equals one; as *eda *gets large it approaches 0.5; as *eda *gets smaller and smaller it approaches 5. The exponent 4 was chosen to approximate the data in [30], figure 2. Note that, in this first model, we are not including explicitly the effects of the autoreceptors on firing rate and dopamine release.

### Storage, release, and reuptake of dopamine

After dopamine is synthesized it is packaged into vesicles by the vesicular monoamine transporter, MAT. We take the *K*_*m *_of the transporter in the literature range (see Table 2) and choose the *V*_*max *_so that the concentration of cytosolic dopamine is in the range 2-3 *μ*M under normal circumstances. The experiments in [65] and the calculations in [66] suggest strongly that there is transport from the vesicles back into the cytosol, either dependent or independent of the MAT. We assume this transport is linear with rate constant, *k*_*out*_, chosen so that the vast majority (i.e., 97%) of the cellular dopamine is in the vesicular compartment. The vesicles take up a significant fraction of the volume terminal, perhaps 1/4 to 1/3 (reference). For simplicity we are assuming that the vesicular compartment is the same size as the non-vesicular cytosolic compartment. This assumption is unimportant since we take the cytosol to be well-mixed and we are not investigating vesicle creation, movement toward the synapic cleft, and recyling where geometry and volume considerations would be crucial.

Vesicular dopamine, *vda*, is put into the synaptic cleft, where it becomes *eda*, by the term *fire*(*t*)(*vda*) in the differential equations for *vda *and *eda *(see above). *fire *is a function of time in some of our *in silico *experiments, for example in Results G where we investigate individual spikes. However, for most of our experiments *fire *= 1 *μ*M/hr, which means that vesicular dopamine is released at a constant rate such that the entire pool turns over once per hour. This is consistent with a variety of experimental results on turnover and we will see in Results C that this choice gives decay curves after *α*-methyl-p-tyrosine (*α*-MT) inhibition of TH that match well the findings of Caron and co-workers [24,25].

Extracellular dopamine has three fates. It is pumped back into the cytosol by the DATs; it is catabolized; it is removed from the system. The parameters for the DATs are taken from the literature. The other two fates are discussed next.

### Metabolism and removal of dopamine

Cytosolic dopamine is catabolized by monoamine oxidase (MAO) and aldehyde dehydrogenase to dihydrophenylacetic acid (*dopac*), which is exported from the neuron and methylated by catecholamine methyl transferase (COMT) to homovanillic acid (*hva*). In this simple model we are not investigating the details of catabolism, only how *cda *is removed from the system. Since the cytosolic dopamine concentration is low (2-3 *μ*M) and the *K*_*m *_for MAO is high (210-230 *μ*M, [67]), the removal of *cda *is basically a linear process that we model by the first order term (*cda*). We choose the rate constant  = 10/*hr *so that the rate of cytosolic catabolism is somewhat less than the synthesis rate of *cda *at steady state. Extracellular dopamine is also catabolized, first by COMT and then by MAO. In this case, we use a Michaelis-Menten formula for this process because the *K*_*m *_of dopamine for COMT is low enough (approximately 3 *μ*M, [68]) that the process saturates in some of our *in silico *experiments in which large amounts of DA are dumped into the extracellular space. The half-life of *hva *is the brain is approximately  hr [69,70], which determines  = 3.45/hr for the removal of *hva *from the system.

In our model the extracellular space is a single compartment. One should think of it as the part of the entire extracellular space corresponding to this particular synapse. Of course, if we had many model synapses, the *eda *from one will diffuse into the extracellular compartment of another (volume transmission). We are assuming for simplicity that the extracellular space is well-mixed, that is, we are ignoring diffusion gradients between different parts of the extracellular space. In fact, Venton et al. [48] have shown using a combination of experiments and modeling that the extracellular space is well-mixed during tonic firing but that substantial gradients exists between "hot spots" of release and reuptake and the rest of the extracellular space during and just after episodes of burst firing. In addition, when SNc projections die, as in Parkinson's disease or in denervation experiments, the terminals will be further apart making it certain that diffusion gradients will play an important role (see the Discussion). The term *k*_*rem*_(*eda*) in the differential equation for *eda *represents removal of *eda *through uptake by glial cells, uptake by the blood, and diffusion out of the striatum. After some experimentation we chose *k*_*rem *_= 400/hr because it gave good fits to the experimental data in [33] discussed in Results B and the experimental data in [24,25] discussed in Results D.

In all cases, steady states or curves showing the variables as functions of time were computed using the stiff ODE solver in MATLAB.

### Steady state concentrations and fluxes

Figure 1 shows the concentrations and velocities at steady state in our model. Only about 10% of the cellular tyrosine input goes to dopamine synthesis with the remainder going to the tyrosine pool (80%) or being catabolized (10%) as seen experimentally [53-55]. Cellular tyrosine itself has a steady state concentration of 126 *μ*M in the model consistent with a large number of experimental observations [58,56,4].

It is known that the cytosolic concentration of dopamine is quite low and the concentration of *l-dopa *is extremely low [3]. In the model, at steady state, *cda *= 2.65 *μ*M and the concentration of *l-dopa *is 0.36 *μ*M, consistent with these observations. It is instructive to look at the flux balance of *cda *in the steady state. 27.3 *μ*M of *cda *are manufactured from tyrosine per hour. 81 *μ*M/hr of dopamine are put into the vesicles by the monoamine transporter and 80.1 *μ*M/hr are put back into the cytosol from the extracellular space by the DATs. Finally, 26.5 *μ*M/hr of dopamine is catabolized in the cytosol.

The largest portion of cellular dopamine is in the vesicles; in our model *vda *= 81 *μ*M at steady state. We assume that at a "normal" firing rate the vesicular contents would be emptied in an hour; that is, *vda *is released into the synaptic cleft at 81 *μ*M/hr. The DATs put most of this *eda *back into the cytosol (80.1 *μ*M/hr), with the remainder being removed (0.81 *μ*M/hr) or being catabolized (.02 *μ*M/hr). We will see below that these velocities are consistent with the half-life measurements of Caron and co-workers [24,25].

## Results

### A. Consequences of substrate inhibition of TH by tyrosine

Tyrosine hydroxylase (TH) converts the amino acid tyrosine into *l-dopa *and *bh*4 into *bh*2; *l-dopa *is then converted by aromatic amino acid decarboxylase into dopamine. Given the dynamic nature of neurons and the importance of dopamine, it is not surprising that TH is regulated by many different mechanisms. TH is inhibited by dopamine itself and is also inhibited by the D2 autoceptors that are stimulated by extracellular dopamine. The effects of these regulations will be discussed below. Here we focus on a third regulation, substrate inhibition of tyrosine hydroxylase by tyrosine [23]. Substrate inhibition means that tyrosine can bind non-enzymatically to TH preventing TH from performing its function of converting tyrosine to *l-dopa*. Substrate inhibition can be competitive (one tyrosine binding to TH makes the catalytic site unavailable to another tyrosine) or uncompetitive (the catalytic site is available to another tyrosine but the enzyme does not perform its catalytic function). Substrate inhibition is not widely recognized as an important regulatory mechanism, though it was proposed by Haldane in the 1930s [71], and it known to have an important homeostatic function in the folate cycle [72]. Figure 2 shows normal Michaelis-Menten kinetics, competitive substrate inhibition, and uncompetitive substrate inhibition. In uncompetitive substrate inhibition the velocity curves rises, reaches a maximum, and then descends to zero because at higher and higher tyrosine concentrations more and more enzyme is bound non-enzymatically to tyrosine.

The velocity curve, figure 2 of [23], shows clearly that the substrate inhibition of TH by tyrosine is uncompetitive and we have chosen our kinetic parameters to match the shape of that curve. The question that we wish to address here is what is the purpose of this substrate inhibition? We will see that it stabilizes vesicular dopamine in the face of variations in tyrosine availability.

**Figure 3:**
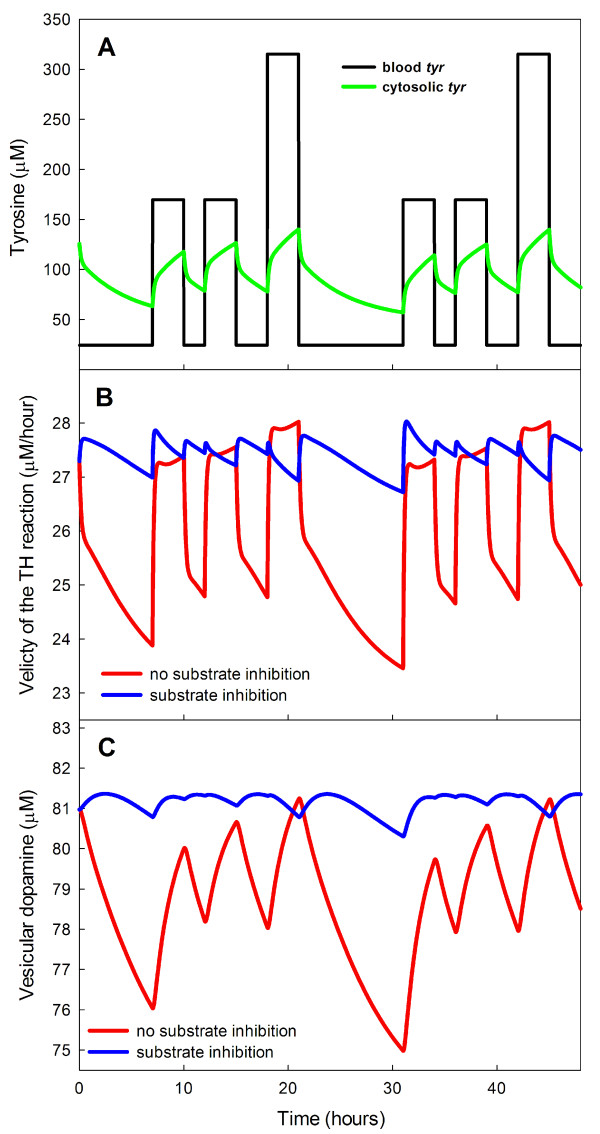
**Dynamic effects of substrate inhibition**. Panel A shows the time courses of blood tyrosine concentration (assumed, see text) and intracellular tyrosine concentration (computed) over a two day period. Panel B shows the time courses of the velocity of the TH reaction over a two day period in response to meals both with and without substrate inhibition. The fluctuations are much smaller when substrate inhibition is present. Panel C shows the time courses of vesicular dopamine in response to meals over a two day period both with and without substrate inhibition. The fluctuations are much smaller when substrate inhibition is present.

It is known [57] that brain tyrosine levels can double after meals, and this implies that tyrosine levels in the blood vary even more dramatically. In our model the average tyrosine level in the blood is 97 *μ*M. We assume that for 3 hours after breakfast and lunch this concentration is multiplied by 1.75 and for three hours after dinner by 3.25. At other times the concentration of blood tyrosine is .25 × 97 = 24.2 *μ*M, which gives a daily average of 97 *μ*M. The blood tyrosine concentrations are shown in Figure 3 along with the cellular tyrosine levels (computed from the model) over a 48 hour period. As found in [57] the intracellular tyrosine levels (roughly the brain levels) vary considerably.

To see the effect of substrate inhibition on the synthesis of L-Dopa by TH, we computed the time courses of the velocity of the TH reaction both with and without substrate inhibition, Panel B of Figure 3. Without substrate inhibition the velocity of the TH reaction varies from 23.5 to 28 *μ*M/hr while in the presence of substrate inhibition the variation ranges only from 27 to 28 *μ*M/hr.

This naturally raises the question of how much the levels of vesicular dopamine vary throughout the day in the two cases. Panel C of Figure 3 shows that substrate inhibition greatly reduces the variation.

We conclude that one important purpose of substrate inhibition is to stabilize the velocity of the TH reaction, and thus the vesicular stores of dopamine, in the face of large variations in tyrosine availability because of meals. The stabilization is a result of the relatively flat velocity curve in a large neighborhood (say 75 *μ*M to 175 *μ*M -see Figure 2) of the normal tyrosine concentration of 126 *μ*M. We note that the non-monotone shape of the velocity curve helps explain some of the unusual relationships between tyrosine levels and dopamine synthesis and release reported in the literature [73,58,56].

### B. The response to prolonged stimulation

**Figure 4:**
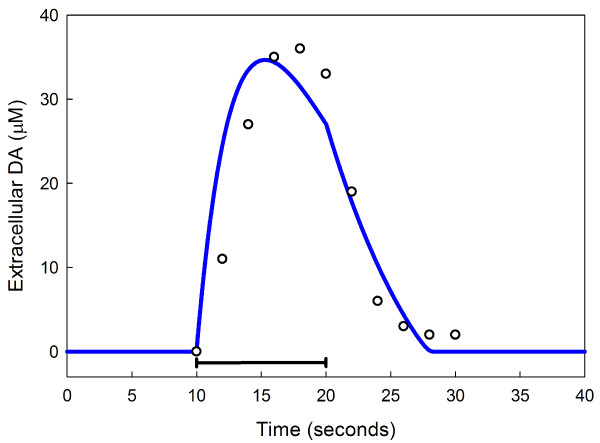
**Extracellular dopamine with 10 seconds of stimulation**. The time course of extracellular dopamine during and after 10 seconds of stimulation (black bar) of dopamin-ergic neurons. Data points (open circles) are redrawn from [33]. The solid line is the model calculation.

In a series of studies and one modeling paper, Justice and co-workers studied the dynamics of extracellular dopamine in dopaminergic neurons in rat brain [34-36,33]. In one experiment they stimulated the ascending projections of SN neurons in the medial forebrain bundle for ten seconds and measured the time course of extracellular dopamine in the striatum. The results of a similar stimulation in our model are shown in Figure 4, which also shows the data in the original experiment. Note that the curve starts to descend before the end of stimulation because of depletion of the reservoir of *vda*. The close match between our model curve and the data suggests that our *V*_*max *_for the DATs (the primary clearance mechanism) is in the right range.

### C. Dopamine turnover in tissues and extracellular space

Over the last 15 years Caron and co-workers have conducted numerous experiments with dominergic neurons. We focus here on the experiments reported in [24], [25] and [74] that compare the behavior of extracellular dopamine and striatal tissue dopamine in wild type mice (WT) and mice that express no DATs at all (DAT^-/-^), the heterozygote (DAT^+/-^), and mice that overexpress the DATs (DAT-tg). The experiments of Caron and co-workers provide an exceptional opportunity to analyze the effects and importance of the DATs.

When we turn off the DATs in our model (by setting the *V*_*max *_to zero), we see changes in steady state values that are qualitatively similar to those seen in [24] and [25] but the magnitudes differ somewhat. The steady state value of *eda *rises by a factor of 10 in the model when the DATs are turned off, while it rises by only a factor of 5 in the DAT^-/- ^mouse. In the model, vesicular dopamine declines from 81 *μ*M to 11 *μ*M when the DATs are turned off, while [24] and [25] report that striatal tissue dopamine in DAT^-/- ^mice is only 1/20 of the value in WT. We modeled the heterozygote (DAT^+/-^) by reducing the *V*_*max *_of the DATs to 1/2 the normal value. The model *eda *increases by 50% compared to WT and *vda *declines by 27%, which is almost exactly the decline in striatal tissue DA reported in DAT^+/- ^mice in ([24], figure 3). In general, one would not expect the model and experimental results to correspond exactly because the DAT^-/- ^and DAT^+/- ^mice have not had their DATs suddenly turned off as we are doing in the model. These mice have lived their whole lives with no or reduced DATs, respectively, so their dopaminergic neurons may differ in other ways from those of the WT mice.

**Figure 5:**
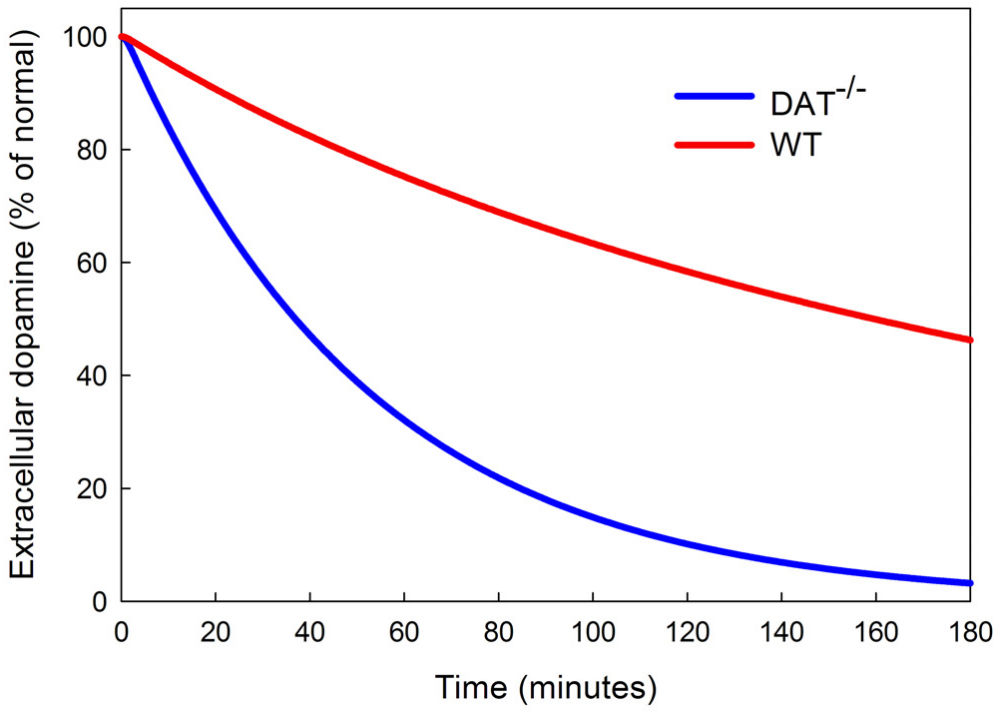
**Inhibition of TH by *α*-MT**. The time course of extracellular dopamine (*eda*) in the model after inhibition of TH by *α*-MT. The half-life of *eda *is 2 hours and 40 minutes for WT mice and 37 minutes for DAT^-/- ^mice.

The studies [24], [25] and [74] report on various experiments that highlight the physiological difference between the WT, DAT^-/-^, and DAT^+/- ^mice. We conducted similar experiments with the model and compared our results to theirs. Figure 1(E,F) of [25] shows the time courses of *eda *for WT and DAT^-/- ^mice after treatment with *α*-methyl-p-tyrosine (*α*-MT), a potent TH blocker. They find half-lives of approximately 2.5 hours for WT and 15-20 minutes for DAT^-/- ^mice. In the model, the half-life of *eda *is 2 hours and 40 minutes for WT mice and 37 minutes for DAT^-/- ^mice; see Figure 5.

**Figure 6:**
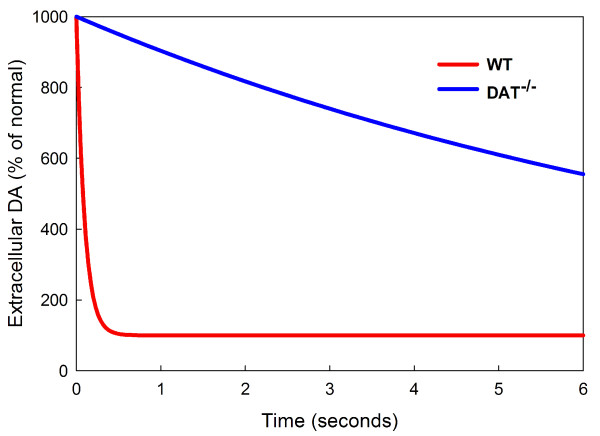
**Clearance of a bolus of eda**. At time *T *= 0 the amount of *eda *is increased by a factor of 10 and the decay back to steady state is shown as a percentage of normal for WT and DAT^-/- ^mice. The half-life of the bolus .067 seconds for WT mice and 6 seconds for DAT^-/- ^mice.

One important focus of the experiments in [24], [25] and [74] is the clearance of *eda *after stimulation. In the model we can test this directly by raising the concentration of *eda *at time *T *= 0 to 10 times normal (for either WT or DAT^-/-^) and measuring the half-life of *eda *as the system relaxes back to equilibrium. See Figure 6.

We find in the model that the half-life of *eda *for WT mice is .067 seconds and the half-life for DAT^-/- ^mice is approximately 6 seconds, giving a ratio of DAT^-/- ^half-life to WT half-life of about 90. Caron and coworkers find similar numbers experimentally except that their ratio is about 300. We note that the ratio is sensitive, of course, to small changes in the small WT half-life that is determined both in experiments and in the model from a very steep curve.

### D. The role of transporter kinetics in the regulation of extracellular dopamine

**Figure 7:**
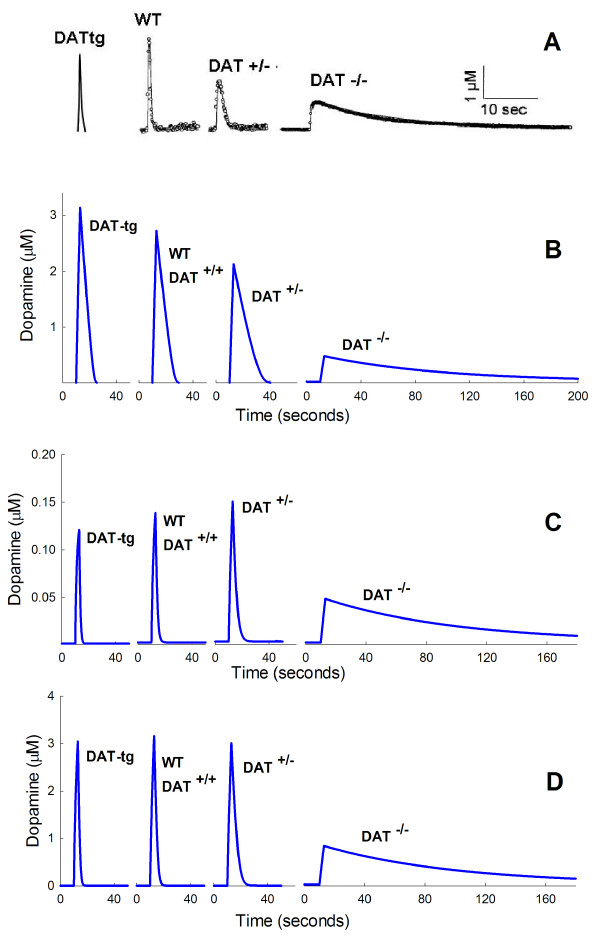
**Time courses of *eda *Panel A shows time courses of *eda *after a pulse of stimulation for DAT-tg, WT, DAT^+/-^, and DAT^-/- ^mice**. The data for DAT-tg mice is taken from figure four B of [74] rescaled to have the same relationship to WT as in that paper. The data on WT, DAT^+/-^, and DAT^-/- ^mice are from figure one of [24]. Panel B shows the time course of *eda *in the model in response to a 300 msec pulse during which the release coefficient *fire *is raised from 1/hr to 900/hr (see Methods) for DAT-tg, WT, DAT^+/-^, and DAT^-/- ^mice. The *V*_*max *_of the DATs is raised 50% for DAT-tg mice, lowered 50% for DAT^+/- ^mice, and set to zero for DAT^-/- ^mice. The *K*_*m *_of the DATs is 0.2 *μ*M. The peaks gets smaller as one moves from DAT-tg to WT to DAT^+/-^. Panel C shows the time course of *eda *in the model in response to a 300 msec pulse during which the release coefficient *fire *is raised from 1/hr to 90/hr (see Methods) for DAT-tg, WT, DAT^+/-^, and DAT^-/- ^mice. The *V*_*max *_values are as in Panel B and the *K*_*m *_of the DATs is 0.2 *μ*M. The peaks now increase as one goes from DAT-tg to WT to DAT^+/-^. Note how narrow the peaks are because the concentrations are lower and the DATs are not saturated. Panel D shows the time course of *eda *in the model in response to a 300 msec pulse during which the release coefficient *fire *is raised from 1/hr to 900/hr (see Methods) for DATg, WT, DAT^+/-^, and DAT^-/- ^mice. The *V*_*max *_vales are as in Panel B. The *K*_*m *_of the DATs is raised to 1.6 *μ*M. The Dat-tg and DAT^+/- ^peaks are both lower than the WT peak as in the experimental data in Panel A.

Some of the most interesting experiments in [24,25] and [74] measure the actual time courses of *eda *in WT, DAT^-/-^, DAT^-/-^, and DAT-tg mice in response to pulse stimulation. Panel A of Figure 7, below, shows a composite of the experimental data taken from [24] and [74]. We were intrigued by the non-monotone character of the peaks. Either more transporters (DAT-tg) or fewer transporters (DAT^+/-^) lowers the *eda *peak compared to wild-type. Our investigations show that this behavior is due to two competing effects.

The first effect is that the amount of dopamine available to be released in response to an external pulse is not strictly proportional to the number of DATs. In the model "the number of DATs" is represented by the *V*_*max *_of the transporter. We increased the *V*_*max *_by 50% for DAT-tg mice compared to wild type, decrease it by 50% for DAT^+/- ^mice, and decrease it to zero for DAT^-/- ^mice. However, *vda*, the pool available for release, has the following model values: *vda *= 98.9 *μ*M (DAT-tg), *vda *= 81 *μ*M (WT), *vda *= 59 *μ*M (DAT^+/-^), *vda *= 11.4 *μ*M (DAT^-/-^). In the model, the amount of dopamine released in response to a pulse small enough not to deplete the *vda *pool very much is proportional to *vda*. Thus more dopamine is released in DAT-tg compared to WT compared to DAT^+/- ^compared to DAT^-/-^. If this were the only effect then one would expect the *eda *peak to be highest for DAT-tg, lower for WT, still lower for DAT^+/-^, and lowest for DAT^-/-^.

The second effect is that if the cell has more DATs, then it should be able to pump the released *eda *back into the cell faster. Thus, if the amount released were the same in each case, we would expect the *eda *peak to be lowest for DATtg, somewhat higher for WT, still higher for DAT^+/-^, and highest for DAT^-/-^. However, as we have seen, the amount released is not the same but decreases as one progresses from DAT-tg to WT to DAT^+/- ^to DAT^-/-^. These are the two competing effects that determine the heights of the peaks. The situation is even more complicated and interesting, however. The *K*_*m *_for the DAT has been measured in a number of experiments. A reasonable range of possible values is 0.2 *μ*M to 2 *μ*M; see [75] and [76]. In the experiments in [24] and [74] the maximal *eda *concentrations were in the range 2-3 *μ*M. This means that if *K*_*m *_= 0.2 then the height of the peak will be highly affected by how much dopamine is released because the DATs are saturated.

In Panel B of Figure 7, we show the time courses of extracellular dopamine in model experiments where *K*_*m *_= 0.2 *μ*M. Notice that the peaks decrease as one goes from DATtg to WT to DAT^+/- ^to DAT^-/-^. In each case the amount of stimulation was the same. This is what one would expect if the first effect, the amount of dopamine released, dominates. To test whether the saturation of the DATs causes this effect we did a model experiment in which the amount of stimulation was reduced to 1/10 of what it was before. The results can be seen in Panel C of Figure 7. At this lower stimulation level, the second effect dominates because the DATs are no longer saturated and the peaks increase as one goes from DAT-tg to WT to DAT^+/-^. Note also how much narrower the peaks are because the DATs are operating on the linear parts of their response curves rather than the saturation part. Panels B and C show that the peaks can be either decreasing or increasing as one proceeds from DAT-tg to WT to DAT^+/-^, depending only on the amount of stimulation. The DAT^-/- ^peak remains lowest because it is not affected by saturation effects on the DATs, since there aren't any.

If we increase the *K*_*m *_of the DATs we will decrease the saturation effects seen in Panel B of Figure 7 and thus the two effects (amount released and rapidity of uptake) should be more evenly balanced. That is indeed the case as shown by Panel D of Figure 7. In Panel D the *K*_*m *_of the DATs has been raised to 1.6 *μ*M and everything else remains the same as in the model experiments shown in Panel B. Now the WT peak is higher than both the DAT-tg peak and the DAT^+/- ^peak. The differences are not great, but the non-monotone effect is clear. As before, the DAT^-/- ^peak is the lowest since so little dopamine is released.

These model experiments show that the relative heights of the peaks depend on the size of the vesicular stores, the number of DATs, their *K*_*m*_s, and the amount of stimulation.

### E. The frequency of stimulation affects passive stabilization

**Figure 8:**
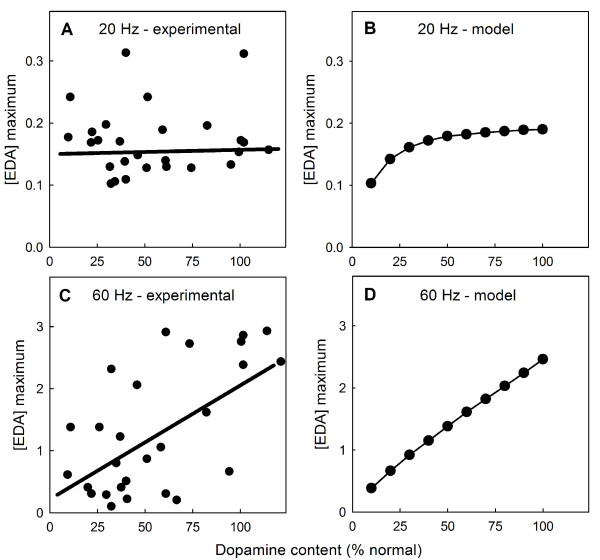
**Denervation affects the peaks of eda in the striatum**. Panels A and C show data and regression lines redrawn from [46], figure 3(a), three (b). In both experiments and the model, tissue dopamine concentration as a percentage of normal is approximately equal to *f*, the fraction of terminals still alive. At 20 Hz stimulation, both the experimental data (Panel A) and the model (Panel B) show that the peaks keep their heights until *f *is very small. This "passive stabilization" is the main focus of [46] and [22]. However, at 60 Hz stimulation, the *eda *peaks decline linearly as *f *declines from one to zero both experimentally (Panel C) and in the model (Panel D).

Bergstrom and Garris [46] measured the time course of extracellular dopamine after two seconds of stimulation in rat striatum after partial denervation. We will let *f *denote the fraction of striatal terminals still alive. With 20 Hz stimulation, the peaks of the resulting *eda *curves are almost independent of *f *until *f *= .15 ([46], figure 3(a), and Panel A of Figure 8). This homeostasis, coined "passive stabilzation" in [77], is the main focus of [46]. By contrast, with 60 Hz stimulation for 2 seconds, the resulting peaks decrease almost linearly as *f *decreases from one towards zero ([46], figure 3(b), and Panel C of Figure 8). Our model shows very similar behavior in both cases (Panels B and D of Figure 8).

Bergstrom and Garris do not explain the reasons why the two cases (20 Hz and 60 Hz) are so different, although the reasons are implicit in their discussions. We give an explanation here using a simple model for *eda *introduced in [44]. As we will see, the difference depends on the *K*_*m *_of the DATs. We denote the (well-mixed) extracellular concentration of dopamine in the striatum by *E*(*t*). We'll ignore removal from the system and catabolism because we are interested in events on a very short time scale. Compared to the concentrations we get after stimulation, *E *starts very small, so we'll assume *E*(0) = 0. Assume that dopamine is released from the cells at a total rate of C/sec for ^*t*^*o *seconds. In [46], *t*_*o *_= 2, so the concentration *E*(*t*) will satisfy the differential equation:

(1)

where we take *K*_*m *_= 0.2 *μ*M as in our large model. For *t *> 2 the *C *isn't there any more but that doesn't affect the maximum concentration of *E*(*t*), which occurs at *t *= 2. We want to calculate *E*(2) and then introduce our scale factor *f *and see how the value scales with *f*.

We will consider two cases. Suppose the release is relatively low as it is in the 20 Hz case where the peak concentrations are (on average) between 0.1 and 0.2 *μ*M. Since the concentrations are below *K*_*m *_we can approximate (1) by:

(2)

We easily solve this differential equation and find



Suppose, now, that only the fraction *f *of cells are still alive. Then *C *is replaced by *fC *and *V*_*max *_is replaced by *fV*_*max*_, so the maximum is:



The shape of the graph of E(2) as a function of *f *depends on the size of . Bergstrom and Garris measured a *V*_*max *_close to 4 *μ*M/sec and *K*_*m *_= 0.2 *μ*M, so  ≈ 40. This means that the graph of *E*(2) will be almost constant as *f *gets smaller from 1. Only when *f *gets very very small will *E*(2) plunge down to zero. This is the constant behavior of the peaks seen in [46], figure 3(a), for 20 Hz stimulation.

In the case of 60 Hz stimulation the peaks are quite high, as much as 3 *μ*M for the intact striatum, far above the *K*_*m *_of the transporters. As long as the concentrations are well above the *K*_*m *_the transporters are saturated and we can approximate (1) by



so the solution at *t *= 2 is:



If only the fraction *f *of cells are left, then,



Thus the maxima should decrease linearly with *f *and that's what Bergstrom and Garris see in their figure 3(b).

This calculation is correct as long as the concentration of *E *stays well above *K*_*m*_. The complete behavior as *f *goes from 1 to 0 in the 60 Hz case should be: first, this linear decrease, then a middle range around the *K*_*m *_value where the rate of decrease is more modest, then a flat plateau as in the first case we considered, until, finally, *E*(2) should plunge to 0 for almost complete denervation. As the ability to take accurate voltametric measurements improves, it will be interesting to see if this prediction is correct.

### F. Homeostatic effects of the autoreceptors

**Figure 9:**
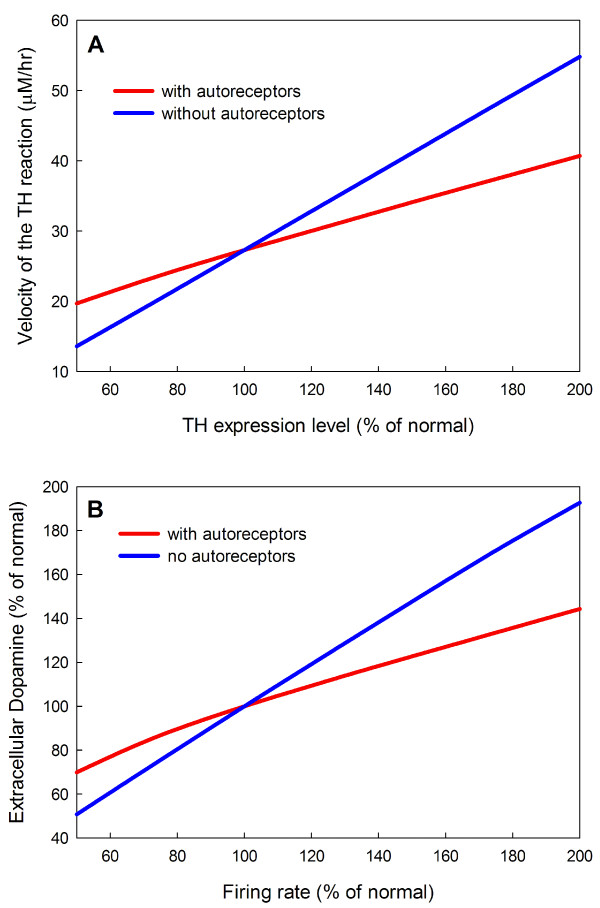
**Homeostatic effects of the autoreceptors**. Panel A shows that the presence of autoreceptors stabilize the velocity of the TH reaction against changes in TH expression level. Panel B shows that the autoreceptors stabilize *eda *against changes in firing rate. *eda *level is shown as a function of firing rate (% percent normal) without autoreceptors present (blue curve) and with autoreceptors (red curve).

It is well established that the expression levels of proteins vary substantially, even in genetically identical cells, and often vary substantially in time in individual cells [78]. Some of this variation is due to control functions in the cell but other variation is due to the stochastic nature of gene expression when small numbers of molecules are involved. The D2 autoreceptors stabilize the velocity of the TH reaction (and therefore vesicular stores) against variation in gene expression level. The mechanism is easy to understand. If the expression of TH drops then the dopaminergic neuron will have less cytosolic and vesicular dopamine and less will be released into the extracellular space. When the concentration of extracellular dopamine drops, the inhibition of TH via the autoreceptors is released, partially compensating for the drop in TH expression. Similarly, if TH is overexpressed, extracellular dopamine rises and the inhibition of TH by the autoreceptors is increased. Panel A of Figure 9 shows how the velocity of the TH reaction depends on the expression level of TH both with and without the autoreceptors. In the presence of autoreceptors the effect of expression level is much milder.

Autoreceptors on the presynaptic membrane upregulate tyrosine hydroxylase(TH) when *eda *drops and downregulate TH when *eda *rises as it will do if the firing rate of the neuron increases [62,61]. Thus, the autoreceptors provide a mechanism whereby the *eda *concentration provides feedback inhibition to TH. The strength of the effect can be seen in Panel B of Figure 9 where *eda *is graphed as a function of firing rate. The *eda *curve is much flatter in the presence of the autoreceptors. We note that the *eda *also affects firing rate directly via somatic autoreceptors [62], but this is not included in our model.

### G. The effect of single action potentials and bursts

It is known [3,4] that there are two typical firing patterns seen in the dopaminergic neurons of the SNc, tonic firing at about 5 Hz and bursts of action potentials with an intraburst frequency of about 15-30 Hz. Dopaminergic neurons respond to reward-related stimuli with increased burst firing [8,79] and burst firing is more effective at raising dopamine levels than tonic firing [80,45]. Recently bursts have been measured in awake, freely-moving animals [81,47,82] in response to rewards and in response to cues for the rewards when the cues have been learned; for a review, see [83].

**Figure 10:**
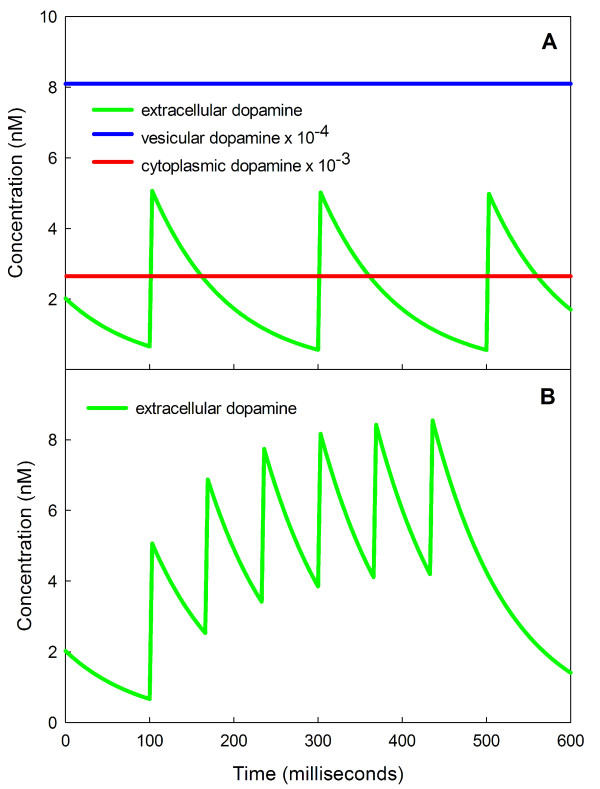
**Bursts increase extracellular dopamine**. Panel A shows the *eda *concentration as a function of time when the tonic firing rate is 5 Hz. The *eda *from the previous action potential is cleared from the extracellular space before the next action potential arrives. Notice that vesicular dopamine and cytosolic dopamine are not noticeably affected on this short time scale. Panel B shows that a short burst of action potentials at 15 Hz raises extracellular dopamine dramatically during the burst. Even a very short term change from tonic firing at 5 Hz to burst firing at 15 Hz produces a large dopamine signal.

We see in our model responses that quite similar to those observed experimentally. Panel A of Figure 10 shows the time course of extracellular dopamine in response to steady firing at 5 Hz. All extra dopamine is cleared from the extracellular space before the next action potential arrives as reported in [76]. Note that on this short time scale cytosolic dopamine and vesicular dopamine remain approximately constant. However, Panel B shows that a burst of action potentials at 15 Hz causes a substantial rise in average *eda *[76]. The model results shown in Figure 10 are similar to the model and experimental results reported in [82], figure 2. Thus, even a very short term shift from tonic firing at 5 Hz to burst firing at 15 Hz produces a large dopamine signal. This shows how sensitive the system is to a brief short-term change in frequency of firing.

**Figure 11:**
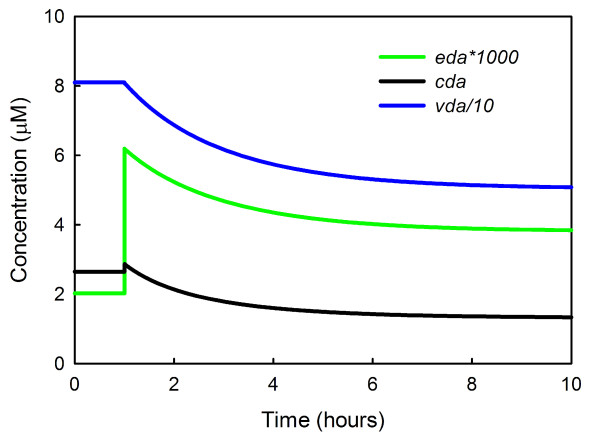
**Habituation to increased firing**. At one hour, the firing rate of the neuron is increased from 5 Hz to 15 Hz and *eda *immediately triples. Then *eda *gradually decreases to an intermediate value since the increased binding of *eda *to the autoreceptors inhibits TH and this causes a gradual decline in vesicular dopamine over a nine hour period. Thus the level of *eda *habituates to the increased firing rate.

However, if firing continues for a long time at 15 Hz, the feedback on TH via the autoreceptors will cause *eda *to decline to an intermediate level, higher than normal but not as high as the short term response. The inhibition of TH by increased binding to the autoreceptors happens quickly, but the resulting decrease in *cda *and *vda *happens slowly over a nine hour period (Figure 11), and this causes a gradual decrease in *eda *even though the firing rate remains elevated. Thus, over the long term, the *eda *concentration gradually habituates to the increased firing rate. It would be interesting to test this prediction of the model experimentally.

## Discussion

The purpose of a mathematical model is not only to summarize or represent the biology that is already known, but to provide a platform for *in silico *experimentation that one can use to explain data, resolve controversies, and try out hypotheses. Our main focus in this paper is to help understand the many homeostatic mechanisms involved in dopamine synthesis, release and reuptake. We have demonstrated that substrate inhibition of tyrosine hydroxylase by tyrosine plays an important role in stabilizing vesicular dopamine against tyrosine fluctuations due to meals. In Section C we studied dopamine turnover and clearance from the extracellular space and compared model results to experimental data. In Section D we used the model to explain features of the time course of extracellualar dopamine observed by Caron and co-worers in DAT knockout and Dat-tg mice. In Section E we showed that the model reproduces the results of Bergstrom and Garris on the different responses to 20 Hz and 60 Hz stimulation and we provide an explanation. In Section F we showed that autoreceptors stabilize extracellular dopamine against changes in expression level of TH and modulate the influence of firing rate on extracellular dopamine concentration. Of course, the purpose of these homeostatic mechanisms is not to make the dopaminergic neuron a fixed object that always responds in the same way. On the contrary, the purpose of the homeostatic mechanisms is keep the neuron poised in the right state, despite environmental fluctuations, so that it can respond appropriately to significant biological signals. Thus, in Section G we showed that the tonic firing rate of 5 Hz keeps extracellular dopamine near normal, but an increase to only 15 Hz in a burst raises extracellular dopamine transiently but significantly. Thus, the neuron is able to send a dopaminergic signal with only a modest and transient increase in firing rate.

Any model includes many oversimplifications. We have not included the details of the use of tyrosine in other metabolic pathways. The processes by which vesicles are created, move to the synapse, and release their dopamine are complicated and interesting [84,65], but are not included in this model. In our model the DATs put released dopamine back into the terminal, but we do not include leakage of cytosolic dopamine through the DATs into the extracellular space. We include in the model the effects of the autoreceptors on dopamine synthesis (via TH) but we do not include explicitly the effects of the autoreceptors on dopamine release and firing rate. Finally, we are focusing on the nerve terminal and on synaptic mechanisms and therefore do not include mechanisms, such as the effects of the autoreceptors on the dendrites and cell body, that operate at the level of the whole cell or between dopaminergic cells.

Such cellular and cell population effects are likely to play important roles in compensatory mechanisms in the case of dopaminergic cell loss. For example, extracellular dopamine concentrations in the striatum are maintained despite massive cell death in the substantia nigra [77,46]. Both passive and active mechanisms including volume transmission, diffusion, and the autoreceptors play a role in this population effect, which we study in [22].

Understanding quantitatively the balance of different mechanisms in dopaminergic cells and cell populations may be crucial for determining proper therapeutic interventions for the dopaminergic dysfunctions mentioned in the introduction. This is a daunting task, complicated by the likelihood of multiple etiologies as well as interactions with nondopaminergic factors [85,86]. Our future goal is to develop the mathematical model so we can use it to explore the variety of proposed hypotheses. We need to understand dopaminergic signaling in the cortex as well as in the basal ganglia in order to understand how the symptoms of Tourette's syndrome arise [87,88]. Both Tourette's and Parkinson's disease press us to study the role of dopamine in shaping activity patterns in cortico-subcortico-cortical circuits and in particular the balance of activity among parallel circuits such as the direct and indirect pathways of the basal ganglia [85,89]. Cognitive dysfunctions including Attention Deficit Hyperactivity Disorder have been attributed to such factors as altered dopamine synthesis [90] and to identified mutations altering the behavior of DATs[91]. Modeling the effects of differential density of DATs [92] or vesicles [84] in dopaminergic neuron populations may help explain why, in the Parkinsonian process of dopaminergic neurodegeneration, neurons projecting to the striatum are characteristically affected earlier than those projecting to other areas such as the Nucleus Accumbens [93].

## Conclusion

Dopaminergic systems must respond robustly to important biological signals such as bursts, while at the same time maintaining homeostasis in the face of normal biological fluctuations in inputs, expression levels, and firing rates. This is accomplished through the cooperative effect of many different homeostatic mechanisms including special properties of tyrosine hydroxylase, the dopamine transporters, and the dopamine autoreceptors. Understanding quantitatively the effects of these homeostatic mechanisms in normal and pathological situations is crucial for the design of therapeutic strategies in a number of neurodegenerative diseases and neuropsychiatric disorders.

## Competing interests

The authors declare that they have no competing interests.

## Authors' contributions

All three authors (JB, MR, HFN) contributed equally to the formulation of the model, the estimation of parameters, experimentation with the model, the biological interpretations and conclusions, and the writing and editing of the manuscript. All authors read and approved the final manuscript.
